# Chloroquine Mediated Modulation of *Anopheles gambiae* Gene Expression

**DOI:** 10.1371/journal.pone.0002587

**Published:** 2008-07-02

**Authors:** Patrícia Abrantes, George Dimopoulos, Ana Rita Grosso, Virgílio E. do Rosário, Henrique Silveira

**Affiliations:** 1 Centro de Malária e Outras Doenças Tropicais- LA/ UEI de Malária, Instituto de Higiene e Medicina Tropical, Universidade Nova de Lisboa, Lisboa, Portugal; 2 W. Harry Feinstone Department of Molecular Microbiology and Immunology, Bloomberg School of Public Health, Johns Hopkins University, Baltimore, Maryland, United States of America; 3 Instituto de Medicina Molecular, Faculdade de Medicina, Universidade de Lisboa, Lisboa, Portugal; 4 University of Cambridge, Department of Oncology, Hutchison-MRC Research Centre, Cambridge, United Kingdom; London School of Hygiene & Tropical Medicine, United Kingdom

## Abstract

**Background:**

*Plasmodium* development in the mosquito is crucial for malaria transmission and depends on the parasite's interaction with a variety of cell types and specific mosquito factors that have both positive and negative effects on infection. Whereas the defensive response of the mosquito contributes to a decrease in parasite numbers during these stages, some components of the blood meal are known to favor infection, potentiating the risk of increased transmission. The presence of the antimalarial drug chloroquine in the mosquito's blood meal has been associated with an increase in *Plasmodium* infectivity for the mosquito, which is possibly caused by chloroquine interfering with the capacity of the mosquito to defend against the infection.

**Methodology/Principal Findings:**

In this study, we report a detailed survey of the *Anopheles gambiae* genes that are differentially regulated by the presence of chloroquine in the blood meal, using an *A. gambiae* cDNA microarray. The effect of chloroquine on transcript abundance was evaluated separately for non-infected and *Plasmodium berghei*-infected mosquitoes. Chloroquine was found to affect the abundance of transcripts that encode proteins involved in a variety of processes, including immunity, apoptosis, cytoskeleton and the response to oxidative stress. This pattern of differential gene expression may explain the weakened mosquito defense response which accounts for the increased infectivity observed in chloroquine-treated mosquitoes.

**Conclusions/Significance:**

The results of the present study suggest that chloroquine can interfere with several putative mosquito mechanisms of defense against *Plasmodium* at the level of gene expression and highlight the need for a better understanding of the impacts of antimalarial agents on parasite transmission.

## Introduction

*Plasmodium* development in the mosquito vector involves several critical steps and the sporogonic cycle needs to be completed successfully within the mosquito for the parasite to be transmitted to the vertebrate host. Mosquito physical and chemical barriers that are represented by the epithelia and the mosquito immune response are important factors contributing to the decrease of parasite numbers during this cycle [1]. Conversely, several external mosquito factors are known to contribute to increasing parasite infectivity, i.e., the ingestion of low-concentration antibodies [2], ingestion of a second blood meal [3], and the presence of the antimalarial drug chloroquine in the blood meal.

Chloroquine ingestion at the time of the blood meal has been associated with an increase in *Plasmodium* infectivity for the mosquito [4]–[6]. In these studies, enhanced infectivity for the mosquito was not associated with increased gametocyte numbers and was observed for several *Plasmodium* species. In an attempt to elucidate further the components and mechanisms underlying this effect, we have used Real-Time PCR to demonstrate for the first time that chloroquine down-regulates some immune-related serine proteases and antimicrobial peptides in *Anopheles gambiae* independently of the presence or absence of *Plasmodium berghei* infection, midgut bacterial flora or the bactericidal effect of chloroquine [6]. We have also recently analyzed the impact of chloroquine on the expression of two previously undescribed *Plasmodium yoelli* genes that are upregulated during transmission in the presence of chloroquine [7]. These results suggests that enhanced infectivity for the mosquito results from chloroquine interference with mosquito defense mechanisms rather than from a direct effect on parasite virulence, given that the chloroquine- mediated down-regulation of immune-related genes was observed both with different doses of the drug and in the absence of infection, while the affected genes have previously been shown to respond positively to *Plasmodium* invasion of midgut cells [8]–[12]. Furthermore, the results suggest that chloroquine acts on the *Anopheles* serine proteases cascade, thereby interfering with immune signal transduction pathways that control the transcription of effector genes. However, the mechanism underlying the down-regulation or the extent of chloroquine interference with the multifaceted defense system of mosquito remains unknown.

Although the use of chloroquine in malaria chemotherapy has been abrogated in many regions due to *Plasmodium falciparum* resistance, chloroquine remains a commonly used agent against *Plasmodium vivax* and other human malarias in most endemic regions [13]. In addition, it has been reported recently that the prevalences of chloroquine resistance markers in *P. falciparum* isolates from some regions of the People's Republic of China and Malawi have decreased or disappeared several years after the discontinuation of chemotherapy [14]–[15]. As a consequence, chloroquine reintroduction is again a subject of debate and understanding the level of chloroquine interference with the mosquito anti-*Plasmodium* defence is emerging as an issue of major importance for the future of malaria treatment strategies. With the present study we aimed to expand our current understanding of the impact of chloroquine on gene expression in the mosquito host by conducting a genome-wide transcript analysis. An *A. gambiae* cDNA microarray platform comprising 20,000 EST clone inserts from various developmental and tissue specific EST libraries [16] was used to analyze mosquito gene expression after exposure to chloroquine under both naïve and malaria- infected conditions.

## Methods

### Biological materials and infections

*Anopheles gambiae s.s.* (SUAKOKO strain) were reared at 25°C and 75% humidity with a 12-hour light/dark cycle and were maintained on a 10% glucose solution. At 5–6 days post-emergence, mosquitoes were blood fed on either: *i*) BALB/c mice (*Mus musculus*) that 17 hours earlier had been treated intraperitonally with a curative dose of chloroquine (50 mg/kg); and *ii)* BALB/c mice infected with *Plasmodium berghei* ANKA clone 2.34 previously treated with 50 mg/kg chloroquine, as described in detail by Abrantes *et al*. [6]. As controls, mosquitoes were similarly fed on: *i*) non-infected BALB/c mice; and *ii*) BALB/c mice infected with *P. berghei* ANKA, respectively. For each group analyzed, mosquitoes were collected 24 hours after blood feeding (the period during which ookinetes invade midgut epithelia, thereby triggering robust mosquito immune response [17]), and batches of 50 midguts were dissected and processed for RNA preparation.

### DNA microarray platform and hybridization

The *Anopheles* cDNA microarray platform (MMC1 or 20K) comprising 20,000 EST clone inserts from various developmental and tissue-specific EST libraries [16] were used for the mosquito gene expression analysis. Total RNA samples from each group of 50 pooled mosquitoes were extracted with TRIzol® (Invitrogen-Life Technologies, Barcelona, Spain), following the manufacturer's instructions. For cDNA synthesis and antisense RNA amplification, the MessageAmp aRNA Kit (Ambion, Huntingdon, UK) was used. Complementary DNA probes were synthesized and labeled with Cy3-dUTP and Cy5-dUTP during the reverse-transcription reaction. Control samples (non-infected untreated and infected untreated mosquitoes, respectively) were labeled with Cy3, and experimental samples (non-infected chloroquine-treated and infected chloroquine-treated mosquitoes, respectively) were labeled with Cy5. Probes were resuspended in hybridization buffer (50% deionized formamide, 6×SSC, 0.5% SDS, 5×Denhardt's reagent) and prehybridized with the arrays in 6×SSC, 0.5% SDS and 1% (vol/vol) BSA at 42°C for 2 hours. The arrays were hybridized overnight at 42°C in humidified hybridization chambers, washed twice in 0.1×SSC, 0.1% SDS for 15 min on an orbital shaker, twice in 0.1×SSC for 5 min and dried. All of the hybridizations (chloroquine-treated mosquitoes vs. untreated mosquitoes or chloroquine-treated infected mosquitoes vs. untreated infected mosquitoes) were performed twice.

### Microarray data analysis

Microarray slides were scanned on a TECAN LS 300 Scanner (Tecan U.S., Inc., Durham, NC) and the images were analyzed with the Chipskipper software version 1.1. (http://www.ansorge-group.embl.de/chipskipper; accessed October 2004). The intensities of the hybridized targets and associated background were estimated. The pre-processing analysis was performed using the Chipskipper software. Spots of poor quality were removed and the background was subtracted. The data were transformed in log2-ratio (M-value) and normalized within-array using the locally weighted linear regression (LOWESS) method [18]. For the between-array normalization, a centering-scale step was used. Only those transcripts that gave regulation signal values concordant for both replicates were considered for the analysis. The final data set was processed using the R limma package [19]–[21]. The genes were ranked according to the *M*-value (mean of the *M-*values for the replicated data sets), and a suitable cut-off (*M*-value >0.58) was determined for genes with expression greater than a 1.5 fold change. The microarray data were submitted to ArrayExpress (http://www.ebi.ac.uk/microarray-as/aer/#ae-main[0]; accession identifier E-MEXP-1201).

Identification of ESTs and assignment of putative gene functions were performed according to AnoEST database version 5(http://web.bioinformatics.ic.ac.uk:8080/AnoEST/anoest.php; [22]), and the Mosquito Genome Database ENSEMBL v.36: Dec 2005 (http://www.ensembl.org/Anopheles_gambiae/index.html). The final expression data for ESTs that belong to the same EST cluster were averaged.

### Quantitative Real-Time PCR analysis

For validation of the microarray results, the same total RNA as that used in the microarray experiments was analyzed. The RNA was treated with DNAse I (Invitrogen- Life Technologies, Barcelona, Spain) and reverse-transcribed with M-MLV-RT (Invitrogen- Life Technologies) in the presence of the Oligo(dt)15 primer (Roche Molecular Biochemicals). Five genes were used for validation of the expression data and included immune genes (serpin, *spi21F;* C-type lectin, *CTL4),* oxidative stress-related genes (cytochrome c oxidase, ENSANGG00000019581; and thioredoxin, *TRX1*), and a protein synthesis-related gene (elongation factor 1 alpha, ENSANGG00000015883). Gene-specific primers were designed with the Primer Express software (Applied Biosystems), and are represented in the supplementary material (Table S1).

Quantitative analysis of the expression of these genes was done by Real-Time PCR with the qPCR core kit for SYBR Green (Eurogentec, S.A., Seraing, Belgium) using the iCycler iQ™ (Bio-Rad). The reactions contained 1x reaction buffer, 200 μM dNTP's, 0.3 μM of each primer, 0.025 U/μl of Hot GoldStar enzyme and 1∶66000 dilution of SYBR Green in a final volume of 20 μl. One microliter (*ca.*0.02%) of cDNA was used as template. The cycle conditions were: initial denaturation at 95°C for 10 minutes, followed by 40 cycles of 95°C for 30 seconds and annealing at specified temperature for 1 minute. The optimized concentrations of MgCl_2_ and annealing temperatures are summarized in Table S1. For all the QRT-PCR assays, the expression levels of the target genes were normalized to the levels of Ribosomal protein *S7* gene utilizing a standard curve method for quantification.

## Results

The effect of chloroquine on *A. gambiae* midgut transcript abundance was assessed by comparing the gene expression levels of *i)* mosquitoes fed on a blood meal that contained chloroquine and those fed on a normal blood meal (*Chl 50*), and *ii)* mosquitoes fed a *P. berghei*-infected blood meal that contained chloroquine and those fed a normal *P. berghei*-infected blood meal (*Chl 50Pb*).

The presence of *P. berghei* parasites committed to sporogonic development was assumed to be the same as in the previous work [6].

**Figure 1:**
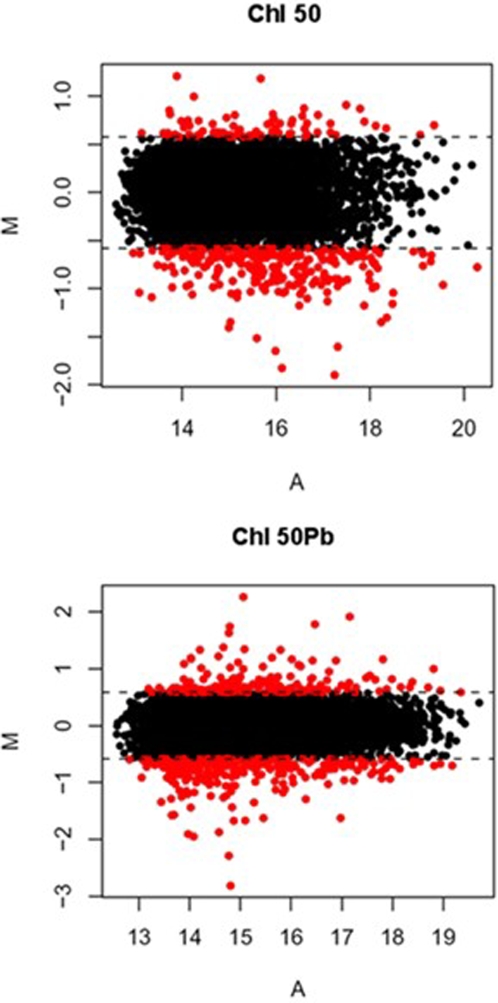
*MA*-plots of the normalized data (*Log2-*ratio plotted against average. Log Intensity) for each experimental group (*Chl 50* and *Chl 50Pb*) with a *Log2* ratio cut-off of 0.58 (i.e., 1.5-fold change). Only those genes that were up- or down-regulated more than 1.5 fold were considered to be differentially expressed.

The analysis of the microarray data revealed that approximately 4.5% of the *A. gambiae* ESTs were up or down-regulated by at least 1.5-fold. The *M*-value and the average log-intensity (A) for the final datasets are shown in Figure 1 by means of an *MA*-plot [23]. Analyses revealed that 126 genes were regulated upon exposure to chloroquine in the absence of infection (*Chl 50*), with 94 genes being down-regulated and 32 genes up-regulated. *P. berghei-* infected mosquitoes (*Chl 50Pb*) showed modulation of expression of 206 genes upon chloroquine exposure: 114 genes were down-regulated and 92 were up-regulated. Complete lists of affected genes are available as supplementary material (Tables S2 and S3).

**Figure 2:**
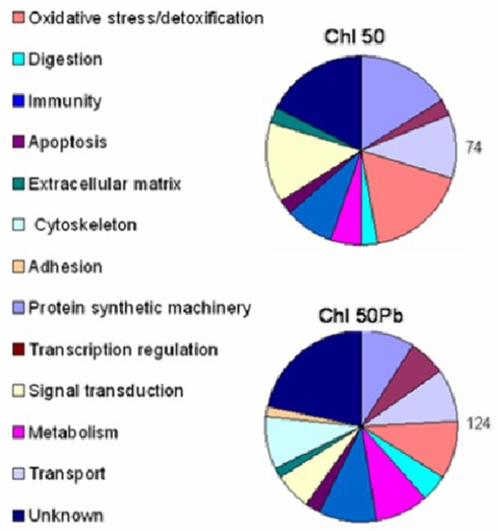
Pie charts indicating the relative proportions of functional gene groups regulated in chloroquine-treated mosquitoes (*Chl 50*) and *P. berghei-*infected chloroquine treated (*Chl 50Pb*) mosquitoes. The functional groups and their corresponding colors in the pie charts are indicated, as well as the total numbers of genes represented in each pie. Ambiguous groups were excluded from this analysis.

The regulated genes represented a variety of functional classes, oxidative stress, digestion, immunity, apoptosis, the extracellular matrix, cytoskeleton, adhesion, the protein synthetic machinery, transcriptional regulation, signal transduction, metabolism and transport, as well as a number of genes of unknown function (Fig. 2, Tables 1 and S4).

**Table 1:** List of genes demonstrating changes in transcript level [fold-increase or decrease (indicated by minus symbol)] in response to the presence of chloroquine in the blood meal of non-infected (*Chl 50*) and *P. berghei-*infected (*Chl 50Pb*) mosquitoes.

EnsEMBL GeneID	ClusterID	Family Description	Gene Name	Chl50	Chl50Pb
**Oxidative Stress**					
ENSANGG00000019348	TCLAG003112	ACYL COA DEHYDROGENASE CHAIN-SPECIFIC MITOCHONDRIAL PRECURSOR			1.5698
ENSANGG00000017291	BBB	ALDO KETO REDUCTASE FAMILY 1 MEMBER		1.5342	
ENSANGG00000018830	TCLAG069348	ADENOSYLHOMOCYSTEINASE		−1.7918	−1.5698
ENSANGG00000009921	TCLAG041742	CYTOCHROME B5-RELATED			−1.7021
ENSANGG00000019581	TCLAG028881	CYTOCHROME C OXIDASE POLYPEPTIDE VA MITOCHONDRIAL PRECURSOR		−1.5566	
ENSANGG00000007821	TCLAG065534	CYTOCHROME C OXIDASE POLYPEPTIDE VIA MITOCHONDRIAL PRECURSOR		−1.5242	
ENSANGG00000001777	TCLAG062787	CYTOCHROME P450			−1.6099
ENSANGG00000020954	TCLAG036258	CYTOCHROME P450		−1.5269	2.0305
ENSANGG00000007941	TCLAG012847	CYTOCHROME P450	*CYP329A1*	−1.5376	
ENSANGG00000023588	TCLAG023097	CYTOCHROME P450	*CYP6AG1*	1.5153	
ENSANGG00000019213	TCLAG003085	CYTOCHROME P450 2			−1.6987
ENSANGG00000023782	TCLAG015411	CYTOCHROME P450 2	*CYP304B1*		1.5940
ENSANGG00000020954	BBB	CYTOCHROME P450 2			2.5172
ENSANGG00000014854	TCLAG067148	PEROXIDASE PRECURSOR			−1.8468
ENSANGG00000014067	TCLAG022599	SULFIDE:QUINONE OXIDOREDUCTASE MITOCHONDRIAL PRECURSOR EC 1			−1.4966
ENSANGG00000007829	TCLAG040227	NADH UBIQUINONE OXIDOREDUCTASE B16 6 SUBUNIT			1.5471
ENSANGG00000015380	TCLAG003027	NADH UBIQUINONE OXIDOREDUCTASE 13 KDA B SUBUNIT			−1.8273
ENSANGG00000001732	TCLAG055584	NADH UBIQUINONE OXIDOREDUCTASE B8 SUBUNIT		−1.5969	
ENSANGG00000013573	TCLAG005781	NADH UBIQUINONE OXIDOREDUCTASE SGDH SUBUNIT MITOCHONDRIAL PRECURSOR		−1.5362	
ENSANGG00000009948	TCLAG063680	DEHYDROGENASE		−1.5414	
ENSANGG00000011473	TCLAG024974	PHOSPHOLIPID HYDROPEROXIDE GLUTATHIONE PEROXIDASE GPX 4		−1.5444	
ENSANGG00000013675	TCLAG026842	SUPEROXIDE DISMUTASE [CU ZN]	*SOD3*	−1.6366	
ENSANGG00000018555	TCLAG045874	THIOREDOXIN 1	*Trx1*	−1.6651	
**Immunity**					
ENSANGG00000019836	TCLAG046632	ANGIOTENSIN-CONVERTING ENZYME PRECURSOR		1.8071	
ENSANGG00000008128	BBB	APOLIPOPROTEIN D PRECURSOR		1.7579	
ENSANGG00000010933	TCLAG018194	SERINE PROTEASE SNAKE PRECURSOR	*CLIPC2*	−1.5518	
ENSANGG00000015439	TCLAG011975	C-TYPE LECTIN	*CTLMA4; CTLMA5*	1.5190	
ENSANGG00000018677	TCLAG002517	C-TYPE LECTIN	*CTL4*		−1.4948
ENSANGG00000009869	TCLAG038131	LEUCINE RICH REPEAT SHOC 2 RAS BINDING SUR 8		−1.5731	
ENSANGG00000019730	TCLAG042327	LEUCINE RICH REPEATS AND IMMUNOGLOBULIN DOMAINS PRECURSOR			1.5522
ENSANGG00000017763	TCLAG035574	AMBIGUOUS	*CLIPA2*		1.5688
ENSANGG00000010766	TCLAG044876	GAMBICIN	*Q9XZN6*		−1.6314
ENSANGG00000015950	TCLAG011952	LYSOZYME C			1.8200
ENSANGG00000011459	TCLAG019577	PEPTIDOGLYCAN RECOGNITION PRECURSOR	*PGRPLB*	−1.6232	−1.6462
ENSANGG00000002044	TCLAG001576	PROPHENOLOXIDASE	*PPO3*		−1.4966
ENSANGG00000016043	TCLAG034331	PROTEASE INHIBITORS PRECURSOR			2.5373
ENSANGG00000013344	TCLAG002324	SERPIN fragment SRPN10	*spi21F*		−1.8327
ENSANGG00000015301	TCLAG065690	SUPPRESSOR OF CYTOKINE SIGNALING	*SOCS*		1.5491
ENSANGG00000011993	TCLAG034787	UNKNOWN	*Transferrin-like Q6VFA3*		−1.6003
**Apoptosis**					
ENSANGG00000016869	TCLAG043243	SPHINGOMYELIN PHOSPHODIESTERASE PRECURSOR		−1.5815	−1.5318
ENSANGG00000024926	TCLAG005775	BACULOVIRAL IAP REPEAT CONTAINING INHIBITOR OF APOPTOSIS	*IAP*	−1.6325	−1.5409
ENSANGG00000006560	TCLAG035786	CASPASE 3 PRECURSOR	*CASPS3*		−1.4986
**Extracellular Matrix**					
ENSANGG00000017996	TCLAG004477	CHITINASE	*O44079*	−1.7583	
ENSANGG00000019293	TCLAG011432	CUTICLE 6 BCNCP14 10		−1.5872	1.5384
ENSANGG00000018296	TCLAG006156	CATHEPSIN B CYSTEINE PROTEINASE PRECURSOR			−1.7229
**Cytoskeleton**					
ENSANGG00000013908	TCLAG059498	ACTIN			−1.7639
ENSANGG00000011396	BBB	ARP2/3 COMPLEX 41 KDA SUBUNIT			−2.3824
ENSANGG00000024209	BBB	PHOSPHATIDYLETHANOLAMINE BINDING PEBP HCNPPP			−1.6014
ENSANGG00000011568	TCLAG031086	RHO GDP DISSOCIATION INHIBITOR			−1.6444
ENSANGG00000008564	TCLAG039850	T COMPLEX 1 SUBUNIT	*chaperonin TCP-1*		−1.5237
ENSANGG00000015160	TCLAG020950	TUBULIN-SPECIFIC CHAPERONE A TUBULIN FOLDING COFACTOR	*TCP-1 cofactor*		−1.9358
ENSANGG00000021087	TCLAG032247	TUBULIN CHAIN	*Tubulin b*		−1.5924
ENSANGG00000014294	TCLAG039279	COLLAGEN ALPHA CHAIN	*Q8T5G4*		1.4956
ENSANGG00000010625	TCLAG007248	DNAJ			1.5818
ENSANGG00000015665	TCLAG020060	MYOSIN LIGHT CHAIN	*Q8T5H8*		2.2895
ENSANGG00000006332	TCLAG020425	TROPOMYOSIN			1.5502
**Adhesion**					
ENSANGG00000021140	TCLAG012865	INNEXIN			1.6587
ENSANGG00000016731	TCLAG064627	UNKNOWN	*Integrin beta*		2.5155

Genes are grouped according to the following functional classes: oxidative stress, immunity, apoptosis, extracellular matrix, cytoskeleton and adhesion.

### Microarrays validation with Quantitative Real-Ttime PCR analysis

Five genes were subjected to QRT-PCR analysis to confirm the robustness of the microarray data (Fig. S1). These genes were selected based on their expression patterns and functions. The genes for cytochrome c oxidase (Ensembl, ENSANGG00000019581), thioredoxin (*TRX1)* and elongation factor 1 alpha (ENSANGG00000015883) were confirmed as being down-regulated in *Chl 50* mosquitoes, while the genes for serpin *sp121F* and C-type lectin *CTL4* were confirmed as being down-regulated in *Chl 50Pb* mosquitoes. Regulation of the elongation factor 1 alpha gene in *Chl 50Pb* mosquitoes was the only exception observed, since the decrease of expression observed by QRT-PCR results contrasted with the weak up-regulation observed in the microarray analysis. This discrepancy may be due to alternative spliced transcripts or cross-hybridization with similar genes. Although not shown in figure S1, another gene confirming microarray results by QRT-PCR was the antimicrobial peptide *gambicin*. As reported previously by our group [6], *gambicin* is down-regulated in infected and non-infected mosquitoes fed on a blood meal containing chloroquine.

### Gene regulation in non-infected A. gambiae transcripts upon chloroquine treatment

We will focus on the description of genes involved in the mosquito defense response. Data concerning other biological processes are detailed in the Supporting information file (Text S1) and in Supplementary Table S4.

#### Oxidative stress/detoxification

Oxidative stress-responsive and detoxification genes represented the major functional classes that responded to chloroquine, with 11 genes being down-regulated and 2 being up-regulated (Table 1). Down-regulated genes coded for four enzymes associated with oxidative phosphorylation (cytochrome c oxidases and NADH ubiquinone oxireductases), a dehydrogenase, an adenosylhomocysteinase, a phospholipid hydroperoxide glutathione peroxidase, the antioxidants thioredoxin 1 (*TRX1*; GenBank accession number: **AL934763**) and zinc-superoxide dismutase (*SOD3*; **BX467242**), and two products associated with detoxification or hormone metabolism (CYP450; **AJ282848**). In contrast, the CYP450 gene (**AL696888**) from the CYP6 gene family was up-regulated, suggesting different cellular functions for the different CYP families. Differential induction of superoxide dismutases (together with catalases) has been associated with broad physiologic differences between refractory and susceptible strains of *A. gambiae* [24].

#### Immunity and apoptosis

Activation of mosquito innate immune response genes has been associated with the ingestion of a blood meal [25]. Twenty-four hours after a blood meal that contained chloroquine, six genes with links to innate immune responses were differentially expressed. Transcripts that code for the peptidoglycan-recognition protein gene PGRPLB (AAAB01008987), the CLIP domain serine protease CLIPC2 (**AL694579**), and a leucine-rich repeat (LRR) domain protein (**AL934484**) were found to be less abundant than in the non-chloroquine-exposed blood-fed controls. On the other hand, transcripts coding for a C-type lectin (**AL694890**), an angiotensin converting enzyme (ACE) precursor (**AL692902**), and the phagocytosis-related apolipoprotein D precursor (**AL932044**) were expressed at higher levels in chloroquine-treated mosquitoes than in the reference controls. Besides being implicated in immunity, C-type (calcium-dependent) lectins and proteins that contain LRRs are involved in a variety of other important biological processes, such as cell adhesion, cell signaling and apoptosis. The transcript levels of an IAP repeat-containing inhibitor of apoptosis (the direct inhibitors of caspases-apoptosis executors), and of a sphingomyelin phosphodiesterase precursor were decreased 24 hours after a blood meal that contained chloroquine. Sphingomyelin phosphodiesterases are implicated in the metabolism of ceramide, a well-defined intracellular mediator of apoptosis in vertebrate defense against intracellular pathogens [26]–[28], and in the responses to *P. berghei* invasion of mosquito midgut cells [29].

### Expression analysis of A. gambiae transcripts following P. berghei infection and chloroquine treatment

The major functional classes of regulated genes in *Chl 50Pb* were oxidative stress, immunity, protein synthetic machinery, transport (see Supplementary Information Text S1), and other genes of unknown function (Fig. 2). Interestingly, the cytoskeletal and adhesion functional classes were differentially regulated by ingestion of an infected blood that contained chloroquine (*Chl 50Pb*) but not by chloroquine in the absence of *P. berghei*; this may be related to the elevated infection levels and associated cytoskeletal reorganizations [16].

#### Oxidative stress

Of the 12 differentially regulated oxidative stress/detoxification genes, 7 were down-regulated and 5 were up-regulated by chloroquine treatment (Table 1). Among the down-regulated genes were those coding for two enzymes associated with oxidative phosphorylation (NADH ubiquinone oxireductase and sulfide:quinone oxidoreductase), an adenosylhomocysteinase, a cytochrome B5-related gene, a peroxidase precursor, and two products associated with the detoxification of harmful substances (CYP450 and CYP450 2). Increased expression levels of similar genes (one CYP450 and two CYP450 2), as well as of an acyl COA dehydrogenase and a NADH ubiquinone oxidoreductase were observed.

Insect peroxidases are involved in detoxification, stabilization of extracellular matrices and insect immunity, mediating the nitration of *Anopheles* midgut cells that are undergoing apoptosis in response to *Plasmodium* invasion [30].

#### Immunity

The presence of 11 gene transcripts with putative immunity functions reinforces our previous data that chloroquine has a significant impact on mosquito immunity-related genes. Among the 11 chloroquine-regulated genes, 2 genes encode pattern recognition receptors, the C-type lectin CTL4 (**BX468701**) and the PGRPLB (AAAB01008987). The antimicrobial peptide *gambicin* gene (**AJ237664**), the *Spi21F*, which encodes a fragment of the serpin *SRPN10* (**BX468769**), prophenoloxidase *PPO3* (**AF004916**), and a gene product of unknown function but with similarities to a transferrin-like protein were found to be repressed in chloroquine-treated infected mosquitoes (*Chl 50Pb*).

In contrast, transcripts that encode a lysozyme, the CLIP domain serine protease CLIPA2 (**BX469333**), a putative serpin, the suppressor of cytokine signaling SOCS (**AL695179**), and a leucine-rich repeat domain protein were induced in chloroquine-exposed infected mosquitoes.

Most of these regulated genes have previously been shown to respond strongly to *P. berghei* invasion of mosquito midgut cells [8]–[12]. The SRPN10 gene, which encoded four alternatively spliced serine protease inhibitors of the serpin superfamily, is a cell-autonomous marker of midgut invasion [11]. The two *A. gambiae* lectins *CTL4* and *CLIPA2* have been shown to act as protective agonists on the development of *Plasmodium* ookinetes to oocysts, since knockdown of either *CTL4* or *CLIPA2/CLIPA5* results in the direct killing of ookinetes through melanization [10], [31]–[32].

#### Apoptosis

Apoptosis-related gene transcripts that were decreased by chloroquine treatment included an IAP (inhibitors of apoptosis; **AL930347**), the *A. gambiae* caspase 3 precursor gene, CASPS3 (**Al934916**), and a sphingomyelin phosphodiesterase precursor gene. Another repressed gene was a cathepsin B cysteine proteinase precursor, which is known to play an important role in extracellular matrix (ECM) remodeling (in addition to being involved in the apoptosis process through cytochrome c release and caspase activation; see below).

#### Cytoskeleton and adhesion

The differential regulation of cytoskeleton- and adhesion-related genes was specific to *Chl 50Pb* and was not observed in non-infected chloroquine-treated mosquitoes. This observation is particularly interesting, since cytoskeletal dynamics and remodeling are considered to be key elements of *P. berghei* invasion of the midgut [16]. Four genes involved in the regulation of actin-cytoskeleton architecture and dynamics were down-regulated by the presence of chloroquine in the blood meal ingested by infected mosquitoes. These genes encode a phosphatidylethanolamine binding protein, an actin, the ARP2/3 Complex 41-kDa subunit (involved in the promotion of actin-filament turnover), and a Rho GDP dissociation inhibitor, which is believed to act as a negative regulator of actin-cytoskeleton remodeling [16]. In addition, three genes implicated in the microtubule cytoskeleton system, including those coding for the tubulin b subunit, TCP-1 chaperonin (involved in tubulin folding), and the tubulin-specific chaperone cofactor, TCP-1 cofactor, were also found to be repressed by chloroquine treatment. The genes that encode the chaperone DNAJ and structural muscle associated-genes (collagen alpha chain, myosin light chain and tropomyosin) were induced. Four genes implicated in cell adhesion and ECM remodeling were also differentially regulated by chloroquine. Of these, the genes that encode innexin (a protein that connects the cytoplasmic compartments of adjacent cells), integrin-beta protein, and the ECM structural protein cuticle 6 bcncp14 were up-regulated whereas the previously discussed cathepsin B cysteine proteinase precursor gene was down-regulated by chloroquine. Cathepsins participate in a variety of processes, including the regulation of immune responses and ECM degradation [33].

### Comparison of differential gene regulation patterns in Chl 50 and Chl 50Pb mosquitoes

The major and most important difference observed between the *Chl 50* and *Chl 50Pb* groups in the mosquito transcriptome analysis was the specific differential regulation of genes involved in the cytoskeleton and cell adhesion in the *Chl 50Pb* group.

Only 13 genes were significantly regulated by both groups, with 5 genes being concordant with respect to regulation pattern and the remaining 8 genes having the opposite pattern of regulation. The effect of chloroquine on the mosquito transcriptome is most strongly supported by concordant genes regulated independently of infection with *P. berghei* parasites. These concordant genes encode the putative immunity-related factors PGRLPB, an IAP, and a sphingomyelin phosphodiesterase precursor.

## Discussion

In the present study, we report the first detailed survey of the *A. gambiae* gene expression response to the antimalarial drug chloroquine under both naïve (*Chl50*) and *Plasmodium-*infected conditions (*Chl50Pb*). While chloroquine affected mosquito genes that are implicated in very diverse functions, the present study focused on those genes that have been linked to innate immunity and processes that are known to be affected by *Plasmodium* infection. Chloroquine induced differential regulation of a plethora of genes implicated in immunity, apoptosis, the cytoskeleton, adhesion, and oxidative stress, confirming our previous findings [6] and further supporting the significant impact that chloroquine has on mosquito anti-*Plasmodium* defense processes.

Several theories have been proposed to explain the mechanism of chloroquine action on biological processes. Most of the described effects of chloroquine on metazoan cells appear to be attributable to alterations of the intravesicular pH, which interferes with membrane and recycling processes [34]–[35]. For example, in human and rat macrophages, the chloroquine mediated-rise in endosomal pH interferes with normal iron metabolism by preventing the endosomal release of iron from the transferrin-transferrin receptor complex, resulting in a decrease in the intracellular concentration of iron [36]–[37]. This decrease in turn affects the functions of several cellular enzymes involved in pathways that lead to the replication of cellular DNA and the expression of various genes. Iron metabolism plays an important role in infection and innate immunity. Changes in iron levels have been implicated in the binding of iron regulatory proteins (IRPs) to iron-responsive elements (IREs), which is part of the insect immune response [38]. The down-regulation of a transferrin-like protein in *Chl 50Pb* mosquitoes is consistent with this process, as transferrin is a eukaryotic iron-binding glycoprotein that controls the levels of free iron in biological fluids [39] and may mediate the effect of chloroquine on mosquito immune responses.

In the present study, most of the immune-related genes that were differentially regulated by chloroquine have previously been shown to respond strongly during *Plasmodium* invasion of the mosquito midgut [8]-[12]. Most of these genes were down-regulated in chloroquine exposed infected mosquitoes which suggest that the immune response is being impaired by chloroquine. Nevertheless, the mosquito *CTL4* gene which acts as a protective factor of the parasite against melanization [10] was also down-regulated in chloroquine-exposed infected mosquitoes. Since *CTL4 is* a parasite agonist, one might expect a decreased level of *P. berghei* infection, in contrast to what was observed. However, Frolet *et al.* [40] have recently proposed that the *A. gambiae* anti-parasitic response has two distinct periods, whereby a preinvasion period characterized by basal immunity regulated by REL1 and REL2 members of the NF-κB transcription factors precedes a post-invasion period characterized by the transcriptional up-regulation of antiparasitic genes. *Plasmodium berghei* development is impaired when the mosquito basal immunity is boosted, and *CTL4* seems to be regulated in a NF-kB-independent manner [40]. A plausible hypothesis is that chloroquine may affect the basal immunity, acting prior to the agonistic action of *CTL4*.

Concordant with the results of previous studies of the rats intestinal mucosa [41], our data suggest that chloroquine decreases apoptosis of *A. gambiae* cells during parasite invasion of the midgut epithelia 24h after the ingestion of an infected blood meal. The down-regulation of important apoptosis-related genes, such as the sphingomyelin phosphodiesterase precursor, which is a mediator of apoptosis that is also known to mediate chloroquine inhibition of NF- κB in epithelial cells [42], suggests that chloroquine may in some way inhibit the apoptosis of midgut cells. Dynamic epithelial rearrangements and the production of cytotoxic radicals associated with apoptosis have been observed during *Plasmodium* midgut invasion [16], [43]–[44]. Therefore, it is tempting to speculate that once apoptosis is inhibited, epithelial repair processes are no longer triggered. Supporting this hypothesis is the observed differential regulation of major ECM-remodeling genes and cathepsin B-related genes. The down-regulation of cathepsin B, cytochrome c or even caspase 3 expression levels in chloroquine-treated mosquitoes reinforces the idea of inactivation of midgut cell apoptosis, and suggests that the cathepsin B-mediated matrix metalloproteinases may be in an inactive state. This repression may also explain the increased infection levels, since apoptosis has been proposed as a mechanism to reduce infection, according to the time-bomb theory [43], in which parasite invasion results in NO production and cell damage. The invaded cells are damaged, become apoptotic, and are extruded from the epithelium by actin constriction. This mechanism allows the midgut to repair the damage caused by parasite invasion.

Regulation of cytoskeleton- and adhesion-related genes in *Chl 50Pb* but not in *Chl 50* mosquitoes is in agreement with the reported designation of cytoskeleton dynamics and remodeling as a local epithelial reaction to midgut invasion by *Plasmodium* [16], [43]–[44], not only as a response to apoptosis but also as antagonists and agonists of parasite development. Genes involved in the regulation of actin-cytoskeleton architecture and dynamics and in the microtubule cytoskeleton system, and probably involved in invasion-related processes, such as the induction of directional lamellipodia and the formation of cytoplasmic lamellar protrusions [16], were down-regulated by chloroquine in *P. berghei-*infected mosquitoes. Knockdown of some of those actin-cytoskeleton-architecture-related genes was shown to increase oocyst numbers [16].

Another highly prevalent group of genes showing regulation by chloroquine was that associated with oxidative stress. This was not surprising, as one of the proposed mechanisms of chloroquine action is interference with reactive oxygen species detoxification systems [reviewed in 45]. Genes represented in this group may also be related to the production of reactive radicals, e.g., nitric oxide (NO), that are associated with mosquito immune response to *Plasmodium* [17].

Taken together, our data show that chloroquine has a significant impact on the transcript abundance of mosquito genes implicated in defense against *Plasmodium,* down-regulating important immune-, apoptosis-, cytoskeleton-, adhesion- and oxidative stress-related genes. This may contribute to the suppression of the well-established defense response system of *Anopheles* vectors in turn leading to the increased infectivity and modulation of parasite gene expression [7] observed in chloroquine-treated infected mosquitoes. As ookinetes invade epithelial cells leading to localized reactions, the all-midgut approach used in the present work might have diluted out alterations of rare transcripts, therefore techniques to identify local foci of gene expression could be used in the future to provide greater insight into the action of chloroquine at cellular level.

Even though we are still not able to dissect the chloroquine mechanism of action underlying this effect, the extent of the impact of chloroquine on mosquito responses is evident from the present study, and it suggests that chloroquine impairs mosquito defenses against *Plasmodium* infection by the potential simultaneous activation of different pathways and modulation of both intravesicular pH-dependent and independent steps. We propose that chloroquine acts by modulating basal immunity, down-regulating apoptosis, and remodeling epithelial cells, thereby permitting more efficient parasite invasion and consequent infectivity changes.

The impact of chloroquine modulation on murine malaria transmission is evident from our studies. It is however unclear whether these results can be extrapolated to human malaria. Differences in the transmission of rodent and human malaria cannot be disregarded, although similar ookinete invasion routes and midgut epithelial responses have been observed for *P. berghei* and *P. falciparum* parasites [43]–[44], [46], and similar anti-*P. falciparum* and anti-*P. berghei* activities have been noted for many immune genes [12], [44], [45]. These similarities, coupled with the assumption that chloroquine is acting primarily through modulation of the mosquito's basal immunity and parasite invasion, suggests that chloroquine could elicit a similar effect on defense responses of mosquitoes infected naturally with human parasites. In this context, it is possible that the effect of chloroquine on the mosquito response to infection has contributed to the rapid dispersal of chloroquine resistance observed in the last 40 years. Chloroquine is still commonly used for the treatment of *P. vivax*, *P. malariae*, *P. ovale*, and *P. knowlesi* infections, and the reintroduction of chloroquine as a potential component of *P. falciparum* combination therapy has been proposed. The present work highlights the need for a better understanding of the impacts of antimalarials on vector and parasite transmission.

## Supporting Information

Table S1 Primers and amplification conditions used in QRT-PCR.
(0.02 MB DOC)

Table S2 Detailed annotations of differentially regulated Chl 50 genes.
(0.07 MB XLS)

Table S3 Detailed annotations of differentially regulated Chl 50Pb genes.
(0.09 MB XLS)

Table S4 List of genes demonstrating transcript level changes in response to presence of chloroquine on the blood meal (cont.).
(0.24 MB DOC)

Figure S1 Validation of DNA Microarray results using Quantitative Real-Time PCR analysis. Effects of chloroquine on the transcript abundances of five genes, as assessed by microarray analysis (in black) and QRT-PCR (grey): cytochrome c oxidase (ENSANGG00000019581), thioredoxin: (TRX1), and elongation factor 1-alpha (ENSANGG00000015883) in non-infected treated mosquitoes (Chl 50); and serpin (spi21F), C-lectin (CTL4), and elongation factor 1-alpha in P. berghei-infected treated mosquitoes (Chl 50Pb). Data were normalized using the A. gambiae ribosomal protein S7 gene expression levels. The Y-axis values represent the mean fold-changes obtained for three independent experiments comparing the chloroquine-treated expression levels to the untreated expression levels.
(0.06 MB TIF)

Text S1 (0.04 MB DOC)
