# Supplementary material for: Chloroquine Mediated Modulation of Anopheles gambiae Gene Expression
Source: PLoS One. 2008 Jul 2;3(7):e2587. doi: 10.1371/journal.pone.0002587 (PMC2432468; doi:10.1371/journal.pone.0002587)
Supplement: Table S1 — Primers and amplification conditions used in QRT-PCR. (0.02 MB DOC) [file pone.0002587.s001.doc]

**Table S1** – Primers and amplification conditions used in QRT-PCR.
