# Supplementary material for: Chloroquine Mediated Modulation of Anopheles gambiae Gene Expression
Source: PLoS One. 2008 Jul 2;3(7):e2587. doi: 10.1371/journal.pone.0002587 (PMC2432468; doi:10.1371/journal.pone.0002587)
Supplement: Table S4 — List of genes demonstrating transcript level changes in response to presence of chloroquine on the blood meal (cont.). (0.24 MB DOC) [file pone.0002587.s004.doc]

**Table S4 -** List of genes demonstrating transcript level changes (fold increase or decrease) in response to presence of chloroquine on the blood meal of non-infected (*Chl 50*) and *P. berghei* infected (*Chl 50Pb*) mosquitoes.

| **EnsEMBL GeneID** | **ClusterID** | **Family Description** | **Gene Name** | **Chl50** | **Chl50Pb** |
| --- | --- | --- | --- | --- | --- |
| **Digestion** |  |  |  |  |  |
| ENSANGG00000016968 | TCLAG005666 | PRECURSOR | *peptidase, chymotrypsin* | -1,5758 |  |
| ENSANGG00000006725 | TCLAG036354 | MALTASE PRECURSOR | *Maltase-like protein Agm2* | -1,5006 |  |
| ENSANGG00000023795 | TCLAG036355 | MALTASE PRECURSOR | *Q17021* |  | 1,5186 |
| ENSANGG00000017677 | TCLAG030514 | AMBIGUOUS | *Serine protease, trypsin family* |  | -1,5086 |
| ENSANGG00000001031 | TCLAG001109 | AMINOPEPTIDASE | *.* |  | 1,5176 |
| ENSANGG00000010695 | TCLAG005230 | TRYPSIN PRECURSOR | *.* |  | -1,6390 |
| ENSANGG00000013313 | TCLAG040943 | TRYPSIN PRECURSOR | *.* |  | 1,9294 |
| ENSANGG00000020716 | TCLAG026785 | PERITROPHIN | *.* |  | 2,2819 |
| **Protein synthetic machinery** |  |  |  |  |  |
| ENSANGG00000006446 | TCLAG013070 | 26S PROTEASOME NON ATPASE REGULATORY SUBUNIT 13 | *.* | -1,5663 |  |
| ENSANGG00000008119 | TCLAG014624 | 26S PROTEASOME NON ATPASE REGULATORY SUBUNIT 8 | *.* | -1,5787 |  |
| ENSANGG00000010856 | TCLAG046977 | 39S RIBOSOMAL L13 MITOCHONDRIAL L13MT | *.* | -1,8237 |  |
| ENSANGG00000008494 | TCLAG016409 | 40S RIBOSOMAL S3A |  | 1,5152 |  |
| ENSANGG00000010054 | TCLAG033476 | 40S RIBOSOMAL S19 | *RS3A_ANOGA* | -1,6608 |  |
| ENSANGG00000014445 | TCLAG027325 | 40S RIBOSOMAL S20 | *.* |  | -2,0878 |
| ENSANGG00000019936 | TCLAG015340 | 60S ACIDIC RIBOSOMAL P2 | *.* | -1,5204 |  |
| ENSANGG00000016725 | TCLAG041568 | 60S RIBOSOMAL L12 |  | -1,5558 |  |
| ENSANGG00000015069 | TCLAG019833 | 60S RIBOSOMAL L35 |  | -1,6483 |  |
| ENSANGG00000018324 | TCLAG020571 | 60S RIBOSOMAL L6 TAX RESPONSIVE ENHANCER ELEMENT BINDING 107 | *.* | -1,7205 |  |
| ENSANGG00000013736 | TCLAG014971 | PROBABLE RIBOSOME BIOGENESIS | *.* | -1,4996 |  |
| ENSANGG00000005711 | TCLAG011685 | RIBOSOME BIOGENESIS BOP1 BLOCK OF PROLIFERATION 1 | *.* |  | -3,1833 |
| ENSANGG00000004572 | TCLAG041337 | RIBOSOME PRODUCTION FACTOR 1 | *.* |  | -1,7525 |
| ENSANGG00000010317 | TCLAG011280 | RIBOSOME RECYCLING FACTOR MITOCHONDRIAL PRECURSOR | *.* |  | -1,5216 |
| ENSANGG00000009701 | TCLAG041859 | RIBONUCLEOSIDE DIPHOSPHATE REDUCTASE LARGE SUBUNIT | *.* |  | -2,5559 |
| ENSANGG00000011536 | TCLAG025188 | PEPTIDYL PROLYL CIS TRANS ISOMERASE NIMA INTERACTING 1 | *.* |  | -1,6433 |
| ENSANGG00000018400 | TCLAG018786 | SIGNAL RECOGNITION PARTICLE 54 KDA |  |  | -1,7306 |
| ENSANGG00000014877 | TCLAG068404 | EUKARYOTIC TRANSLATION INITIATION FACTOR 4B | *.* |  | -1,9630 |
| **EnsEMBL GeneID** | **ClusterID** | **Family Description** | **Gene Name** | **Chl50** | **Chl50Pb** |
| ENSANGG00000016354 | TCLAG054491 | EUKARYOTIC PEPTIDE CHAIN RELEASE FACTOR SUBUNIT 1 | *.* | -1,5432 |  |
| ENSANGG00000015883 | TCLAG011974 | ELONGATION FACTOR 1 ALPHA | *.* | -1,7701 | 1,5929 |
| ENSANGG00000017200 | TCLAG043094 | 39S RIBOSOMAL L28 MITOCHONDRIAL | *.* |  | 1,5845 |
| ENSANGG00000017176 | TCLAG043043 | METHIONINE AMINOPEPTIDASE 2 | *.* |  | 1,7271 |
| **Transcription regulation** |  |  |  |  |  |
| ENSANGG00000016741 | TCLAG064616 | HIRA INTERACTING 5 |  | 1,5004 |  |
| ENSANGG00000018166 | TCLAG021250 | PRE SPLICING FACTOR 18 HOMOLOG | *.* | -1,5049 |  |
| ENSANGG00000016011 | TCLAG013501 | NUCLEOSOME ASSEMBLY 1 | *.* |  | -1,6554 |
| ENSANGG00000015711 | TCLAG028202 | NUCLEOTIDE BINDING | *.* |  | -1,5166 |
| ENSANGG00000000004 | BBB | HISTONE H2A | *P90675* |  | -1,5379 |
| ENSANGG00000019617 | TCLAG013074 | HISTONE DEACETYLASE COMPLEX SUBUNIT | *.* |  | 1,5036 |
| ENSANGG00000016971 | TCLAG044491 | CORNICHON | *.* |  | 1,7055 |
| ENSANGG00000015901 | TCLAG005893 | DNA DIRECTED RNA POLYMERASES III 12.5 KDA POLYPEPTIDE | *.* |  | 1,5006 |
| ENSANGG00000007764 | TCLAG031729 | TRANSCRIPTION FACTOR V MAF | *.* |  | 3,7698 |
| ENSANGG00000017570 | TCLAG045803 | TRANSCRIPTION INITIATION FACTOR IIB | *.* |  | 1,5771 |
| **Signal Transduction** |  |  |  |  |  |
| ENSANGG00000009932 | TCLAG041698 | ADENOSINE KINASE | *.* | -1,7758 |  |
|  | TCLAG028588 | CELL DIVISION KINASE | *.* | -1,7196 |  |
| ENSANGG00000023550 | TCLAG004963 | CYCLIN T1 | *.* | 1,6188 |  |
| ENSANGG00000002696 | TCLAG000998 | LINGERER |  | 1,6455 |  |
| ENSANGG00000008239 | TCLAG012194 | KINASE | *.* | 1,6148 |  |
| ENSANGG00000018432 | TCLAG040025 | MYOTUBULARIN |  | -1,4970 |  |
| ENSANGG00000002350 | TCLAG021784 | CALCIUM/CALMODULIN DEPENDENT KINASE TYPE II CHAIN | *.* | 1,6731 |  |
| ENSANGG00000014380 | TCLAG022082 | CELLULAR RETINALDEHYDE BINDING CRALBP | *.* | 1,6978 |  |
| ENSANGG00000012065 | TCLAG045418 | CELLULAR RETINALDEHYDE BINDING CRALBP | *.* | -1,5828 |  |
| ENSANGG00000020291 | TCLAG039617 | CELLULAR RETINALDEHYDE BINDING CRALBP | *.* | 2,2726 | 1,5196 |
| ENSANGG00000008183 | TCLAG007050 | CELLULAR RETINALDEHYDE BINDING CRALBP |  |  | 1,5076 |
| ENSANGG00000007567 | TCLAG031632 | ADENYLATE CYCLASE TYPE | *.* |  | 1,7865 |
| ENSANGG00000017933 | TCLAG024627 | GTP BINDING | *.* |  | -1,7806 |
| ENSANGG00000019751 | TCLAG040198 | PROHIBITIN B CELL RECEPTOR ASSOCIATED | *.* |  | 1,5834 |
| ENSANGG00000019233 | TCLAG007840 | SERINE THREONINE KINASE RECEPTOR ASSOCIATED UNR INTERACTING | *.* |  | -1,5553 |
| ENSANGG00000010083 | TCLAG041798 | SERINE/THREONINE PHOSPHATASE | *.* |  | -1,7226 |
| **EnsEMBL GeneID** | **ClusterID** | **Family Description** | **Gene Name** | **Chl50** | **Chl50Pb** |
| ENSANGG00000018496 | TCLAG045883 | SODIUM/POTASSIUM TRANSPORTING ATPASE BETA CHAIN | *.* |  | 1,6982 |
| **Metabolism** |  |  |  |  |  |
| ENSANGG00000010796 | TCLAG011294 | 3 KETOACYL COA THIOLASE PEROXISOMAL PRECURSOR | *.* | -1,8127 |  |
| ENSANGG00000011890 | TCLAG042087 | PYRUVATE DEHYDROGENASE KINASE | *.* | 1,7357 |  |
| ENSANGG00000006645 | TCLAG035591 | ORNITHINE DECARBOXYLASE | *.* | -1,5895 |  |
| ENSANGG00000010076 | TCLAG047559 | ORNITHINE DECARBOXYLASE ANTIZYME |  |  | 1,5730 |
| ENSANGG00000001295 | TCLAG068876 | UBIQUITIN CARBOXYL TERMINAL HYDROLASE 16 | *.* | 1,5815 |  |
| ENSANGG00000019219 | TCLAG002884 | UBIQUITIN CARBOXYL TERMINAL HYDROLASE ISOZYME L5 | *.* |  | -1,6003 |
| ENSANGG00000022924 | TCLAG029224 | ALKALINE PHOSPHATASE PRECURSOR | *.* |  | -1,8056 |
| ENSANGG00000019643 | TCLAG014536 | HDHPR QUINOID DIHYDROPTERIDINE REDUCTASE | *.* |  | -1,5036 |
| ENSANGG00000014206 | TCLAG045056 | FATTY ACID SYNTHASE | *.* |  | -1,7548 |
| ENSANGG00000011118 | BBB | OXYSTEROL BINDING RELATED 5 | *.* |  | -1,7795 |
| ENSANGG00000025162 | TCLAG002844 | OXYSTEROL BINDING RELATED | *.* |  | -1,5615 |
| ENSANGG00000013276 | TCLAG030896 | TREHALASE PRECURSOR |  |  | -1,6477 |
| ENSANGG00000019664 | TCLAG013071 | TRIACYLGLYCEROL LIPASE GASTRIC PRECURSOR | *.* |  | -2,2207 |
| ENSANGG00000007592 | TCLAG036733 | GLYCOGEN PHOSPHORYLASE FORM | *.* |  | 1,8092 |
| ENSANGG00000016255 | TCLAG023105 | PHOSPHOENOLPYRUVATE CARBOXYKINASE [GTP] | *.* |  | 1,5257 |
| **Transport** |  |  |  |  |  |
| ENSANGG00000014104 | TCLAG031243 | SULFATE TRANSPORTER |  | -1,5905 | 1,5116 |
| ENSANGG00000020745 | TCLAG045608 | ATP BINDING CASSETTE SUB FAMILY G MEMBER 1 | *.* |  | -2,0399 |
| ENSANGG00000008301 | TCLAG014782 | ATP BINDING CASSETTE SUB FAMILY F MEMBER 2 | *.* | 1,5374 |  |
| ENSANGG00000013134 | TCLAG029227 | PHOSPHATIDYLINOSITOL N ACETYLGLUCOSAMINYLTRANSFERASE SUBUNIT P | *.* |  | -1,5440 |
| ENSANGG00000009787 | TCLAG045292 | P MELANOCYTE SPECIFIC TRANSPORTER PINK EYED DILUTION | *.* |  | 1,6088 |
| ENSANGG00000010074 | TCLAG033378 | SYNAPTIC VESICLE 2 SV2 | *.* |  | 1,7806 |
| ENSANGG00000003042 | TCLAG008359 | SOLUTE CARRIER FAMILY 2 FACILITATED GLUCOSE TRANSPORTER MEMBER | *.* |  | 1,6543 |
| ENSANGG00000017267 | TCLAG023398 | SOLUTE CARRIER FAMILY 2 FACILITATED GLUCOSE TRANSPORTER MEMBER | *.* | -1,6102 |  |
| ENSANGG00000016914 | TCLAG068296 | SODIUM DEPENDENT TRANSPORTER | *.* |  | -1,8851 |
| ENSANGG00000015686 | TCLAG033297 | SODIUM DEPENDENT TRANSPORTER | *.* |  | -1,7294 |
| ENSANGG00000004000 | TCLAG044706 | SODIUM/HYDROGEN EXCHANGER SOLUTE CARRIER FAMILY 9 MEMBER | *Q6Y8G3* |  | -1,6024 |
| ENSANGG00000010760 | TCLAG018000 | SODIUM DEPENDENT PHOSPHATE TRANSPORTER 1 | *.* | 1,5076 |  |
| ENSANGG00000010225 | TCLAG028448 | SODIUM/POTASSIUM/CALCIUM EXCHANGER RETINAL | *.* | -1,5840 |  |
| ENSANGG00000014297 | TCLAG039233 | TRANSPORT SEC61 ALPHA SUBUNIT | *sec61* | -1,8658 |  |
| **EnsEMBL GeneID** | **ClusterID** | **Family Description** | **Gene Name** | **Chl50** | **Chl50Pb** |
| ENSANGG00000020729 | TCLAG032767 | VACUOLAR ATP SYNTHASE 16 KDA PROTEOLIPID SUBUNIT | *.* | -1,8945 |  |
| ENSANGG00000014141 | TCLAG015572 | VACUOLAR ATP SYNTHASE CATALYTIC SUBUNIT A UBIQUITOUS | *.* | 1,6236 |  |
| ENSANGG00000008028 | TCLAG060732 | VACUOLAR ATP SYNTHASE SUBUNIT D | *.* |  | 1,5297 |
| ENSANGG00000000991 | TCLAG001157 | TRANSMEMBRANE 9 SUPERFAMILY MEMBER PRECURSOR | *.* |  | 1,5594 |
| **Unknown** |  |  |  |  |  |
| ENSANGG00000019183 | TCLAG002859 | UNKNOWN | *.* |  | -3,0876 |
| ENSANGG00000010673 | TCLAG005181 | UNKNOWN | *.* |  | -2,3588 |
| ENSANGG00000013822 | TCLAG032340 | UNKNOWN | *.* |  | -1,9747 |
| ENSANGG00000021906 | TCLAG029627 | UNKNOWN | *.* |  | -1,8566 |
| ENSANGG00000010245 | TCLAG067603 | UNKNOWN | *.* |  | -1,7340 |
| ENSANGG00000008582 | TCLAG037937 | UNKNOWN | *.* |  | -1,6920 |
| ENSANGG00000024988 | TCLAG016860 | UNKNOWN | *.* |  | -1,6808 |
| ENSANGG00000015100 | TCLAG022409 | UNKNOWN | *.* |  | -1,6174 |
| ENSANGG00000012614 | TCLAG023875 | UNKNOWN | *.* |  | -1,5605 |
| ENSANGG00000014006 | TCLAG044751 | UNKNOWN | *.* |  | -1,5522 |
| ENSANGG00000014630 | TCLAG003989 | UNKNOWN | *.* |  | -1,5502 |
| ENSANGG00000012397 | TCLAG063786 | UNKNOWN | *.* |  | -1,5116 |
| ENSANGG00000011561 | TCLAG036142 | UNKNOWN | *.* |  | -1,5026 |
| ENSANGG00000015259 | TCLAG010203 | UNKNOWN | *.* |  | -1,5016 |
| ENSANGG00000019914 | TCLAG002430 | UNKNOWN | *.* |  | 1,4986 |
| ENSANGG00000013187 | TCLAG040894 | UNKNOWN | *.* |  | 1,5036 |
| ENSANGG00000022827 | TCLAG034149 | UNKNOWN | *.* |  | 1,5086 |
| ENSANGG00000017743 | TCLAG011184 | UNKNOWN | *.* |  | 1,5126 |
| ENSANGG00000008259 | TCLAG021907 | UNKNOWN | *.* |  | 1,5166 |
| ENSANGG00000016281 | TCLAG066151 | UNKNOWN | *.* |  | 1,5216 |
| ENSANGG00000014249 | TCLAG045065 | UNKNOWN | *.* |  | 1,5379 |
| ENSANGG00000017397 | TCLAG067257 | UNKNOWN | *.* |  | 1,5563 |
| ENSANGG00000009124 | TCLAG020034 | UNKNOWN | *.* |  | 1,6174 |
| ENSANGG00000014136 | TCLAG046196 | UNKNOWN | *.* |  | 1,6379 |
| ENSANGG00000010881 | TCLAG025133 | UNKNOWN | *.* |  | 1,7537 |
| ENSANGG00000008653 | TCLAG045707 | UNKNOWN | *.* |  | 1,7607 |
| ENSANGG00000016732 | TCLAG068657 | UNKNOWN | *.* |  | 2,0962 |
| **EnsEMBL GeneID** | **ClusterID** | **Family Description** | **Gene Name** | **Chl50** | **Chl50Pb** |
| ENSANGG00000009757 | TCLAG012846 | UNKNOWN | *.* | -2,0591 |  |
| ENSANGG00000016619 | TCLAG064856 | UNKNOWN | *.* | -1,6606 |  |
| ENSANGG00000009583 | TCLAG012443 | UNKNOWN | *.* | -1,6104 |  |
| ENSANGG00000007851 | TCLAG040229 | UNKNOWN | *.* | -1,6007 |  |
| ENSANGG00000014439 | TCLAG015021 | UNKNOWN | *.* | -1,5835 |  |
| ENSANGG00000014986 | TCLAG012669 | UNKNOWN | *.* | -1,5614 |  |
| ENSANGG00000014839 | TCLAG068407 | UNKNOWN | *.* | -1,5297 |  |
| ENSANGG00000011280 | TCLAG031525 | UNKNOWN | *.* | -1,5153 |  |
| ENSANGG00000011561 | TCLAG036141 | UNKNOWN | *.* | -1,5080 |  |
| ENSANGG00000019159 | TCLAG005002 | UNKNOWN | *.* | -1,5059 |  |
| ENSANGG00000009273 | TCLAG036879 | UNKNOWN | *.* | -1,4992 |  |
| ENSANGG00000019939 | TCLAG010960 | UNKNOWN | *.* | -1,4984 |  |
| ENSANGG00000008170 | TCLAG008046 | UNKNOWN | *.* | 1,5337 |  |
|  |  |  |  |  |  |

Genes were grouped into the following functional classes: digestion, protein synthetic machinery, transcription regulation, signal transduction, metabolism, transport, as well as a number of unknown genes.
