# Supplementary figures and images for: Chloroquine Mediated Modulation of Anopheles gambiae Gene Expression
Source: PLoS One. 2008 Jul 2;3(7):e2587. doi: 10.1371/journal.pone.0002587 (PMC2432468; doi:10.1371/journal.pone.0002587)

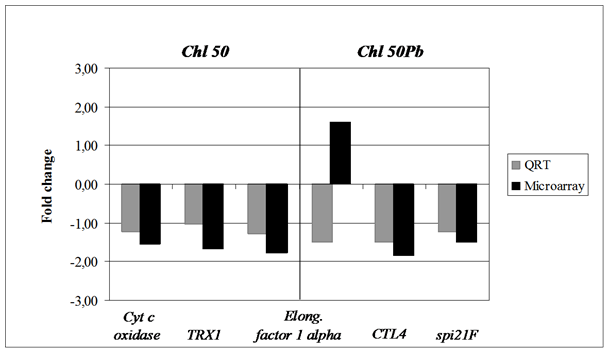

Supplement: Figure S1 — Validation of DNA Microarray results using Quantitative Real-Time PCR analysis. Effects of chloroquine on the transcript abundances of five genes, as assessed by microarray analysis (in black) and QRT-PCR (grey): cytochrome c oxidase (ENSANGG00000019581), thioredoxin: (TRX1), and elongation factor 1-alpha (ENSANGG00000015883) in non-infected treated mosquitoes (Chl 50); and serpin (spi21F), C-lectin (CTL4), and elongation factor 1-alpha in P. berghei-infected treated mosquitoes (Chl 50Pb). Data were normalized using the A. gambiae ribosomal protein S7 gene expression levels. The Y-axis values represent the mean fold-changes obtained for three independent experiments comparing the chloroquine-treated expression levels to the untreated expression levels. (0.06 MB TIF) [file pone.0002587.s005.tif]
