# Supplementary material for: Chloroquine Mediated Modulation of Anopheles gambiae Gene Expression
Source: PLoS One. 2008 Jul 2;3(7):e2587. doi: 10.1371/journal.pone.0002587 (PMC2432468; doi:10.1371/journal.pone.0002587)
Supplement: Text S1 — (0.04 MB DOC) [file pone.0002587.s006.doc]

**Text S1**

**Results**

*Expression analysis of non-infected A. gambiae transcripts after chloroquine treatment:*

The functional classes more represented at the *Chl 50* group were those that include genes involved in oxidative stress, protein synthesis machinery, transport, signal transduction and unknown function (Fig. 2).

*Protein synthesis*

Twelve genes coding for products associated with protein synthesis, folding and modification (ribosomal subunits, ribosome biogenesis factors, elongation factor 1 alpha (**BX469675**), and eukaryotic peptide chain release factor subunit 1) and transcription regulation (pre splicing factor 18 homolog) were significantly reduced in expression (Table S4). In contrast solely two genes associated protein production machinery showed increased expression, one coding for 40S ribosomal S3A subunit and the other coding for Hira interacting 5, an histone-interacting protein.

*Digestion and Extracellular matrix*

Twenty-four hours following a blood meal containing chloroquine, the differential expressed transcripts that were involved in blood digestion showed a decreased level of transcription. These included a peptidase digestive enzyme and the Maltase-like protein Agm2 which is related to sugar meal digestion [1].

Transcripts coding for midgut structural proteins, such as those coding for a cuticle 6 and a protein similar to chitinase had also decreased expression.

*Signal transduction*

Genes coding for two kinases (adenosine kinase and cell division kinase), the phosphatase myotubularin and one cellular retinaldehyde binding protein (CRALBP) involved in mosquito vision were down-regulated, contrasting with the increase of expression of two genes coding for a similar protein (CRALBP), two kinases (kinase and a calcium/calmodulin dependent kinase, involved in calcium signaling), one gene associated with cell-cycle control (cyclin T1) and transcripts coding for a lingerer.

*Metabolism*

Transcripts coding for four enzymes probably associated with energy metabolism were differentially regulated, indicating decrease expression of genes coding for the 3 ketoacyl COA thiolase precursor involved in lipid synthesis and the ornithine decarboxylase enzyme involved in polyamine biosynthesis. On the other hand, increase expression of transcripts coding for an ubiquitin carboxyl hydrolase and the glycolytic pathway enzyme pyruvate dehydrogenase kinase were observed.

*Transport*

Eight genes with possibly implication in transport process, namely ion and water transport, were differentially regulated by chloroquine. Genes coding for a sulfate transporter, the solute carrier family 2 facilitated glucose transporter, a sodium/potassium/calcium exchanger, the vacuolar ATP synthase 16 kda proteolipid subunit and the sec61 alpha subunit transporter (**AJ419878**) involved in protein translocation, were down-regulated. Among the three transporters up-regulated by the presence of chloroquine on the blood meal, genes coding for an ATP binding cassette sub family F member, a sodium dependent phosphate transporter and a vacuolar ATP synthase catalytic subunit were found.

*Expression analysis of A. gambiae transcripts following P. berghei infection and chloroquine treatment:*

*Protein synthesis*

Among the differentially regulated genes, eight gene products coding components of protein synthesis machinery (one ribosomal subunit, three products associated with ribosome biogenesis, a ribonucleoside diphosphate reductase, a peptidyl prolyl cis trans isomerase, a translation initiation factor and a signal recognition particle) were down-regulated and three up-regulated by chloroquine treatment (Table S4). The up-regulated genes code for an elongation factor, a ribosomal subunit and a methionine aminopeptidase.

*Digestion*

The ingestion of a blood meal containing chloroquine by infected mosquitoes decreased transcription of two gene products involved in protein degradation, a trypsin precursor and a trypsin serine protease. On the other hand, transcription of products coding for an aminopeptidase, a trypsin precursor and the Maltase precursor Q17021 (**X87410**) increased in expression. Also observed was the induction of the structural midgut epithelium perithrophin gene usually linked to the formation of perithrophic matrix immediately after the ingestion of blood meals [2].

*Transcription regulation*

Expression of five genes coding for products associated with transcription and nuclear regulation, such as two transcription factors, an histone, a DNA directed RNA polymerase and a gene coding for cornichon, was significantly increased while genes coding for two nuclear genes and the histone P90675 (**BX468264**) decreased in expression in *Chl 50Pb*.

*Signal transduction*

Presence of chloroquine on infected mosquito’s blood meal increased transcription of five genes associated with signal transduction, more specifically, transcripts associated with cell-cycle control (prohibitin B cell receptor), regulation of intracellular ion levels (sodium/potassium transporting ATPase) and vision (CRALBP). Transcripts coding for an adenylate cyclase type protein were also induced. Decreased transcription was observed in genes coding for a serine threonine kinase receptor, as well as for genes involved in protein phosphorylation pathways (serine/threonine phosphatase and a GTP binding protein).

*Metabolism*

Also associated with blood meal and its contribution to energy needs of female mosquitoes, several transcripts involved in insect adult energy metabolism were differentially regulated by chloroquine. These included down-regulation of two enzymes involved in lipid metabolism (fatty acid synthase and triacylglycerol lipase), the principal energy source for mosquitoes when at rest [3], two genes involved in Golgi secretion (oxysterol binding related), one gene involved in glycolysis-related processes (trehalase precursor) and genes coding for a ubiquitin carboxyl hydrolase, an alkaline phosphatase precursor and a quinoid dihydropteridine reductase. Increased expression was observed in transcripts associated with glycogen degradation (glycogen phosphorylase), gluconeogenesis and glycolysis (phosphoenolpyruvate carboxykinase [GTP]), the major pathway by which carbohydrates are degraded in insects for energy release and mostly related to energy source for flight [3]. Transcripts involved in polyamine biosynthesis coding for an ornithine decarboxylase antizyme were also increased in expression.

*Transport*

Genes coding for the transporters ATP binding cassette sub family G member, phosphatidylinositol N acetylglucosaminyltransferase, sodium dependent transporters and sodium/hydrogen exchanger solute carrier (Q648G3; **AL696382**) were down-regulated. Genes coding for a sulfate transporter, a synaptic vesicle 2, a solute carrier family 2 facilitated glucose transporter member, a vacuolar ATP synthase subunit D protein and a transmembrane 9 superfamily member precursor were more expressed after the blood meal containing chloroquine, as was the gene coding for a P-melanocyte specific transporter.

**Supporting References**

[1] Zheng L, Whang LH, Kumar V, Kafatos FC (1995) Exp Parasitol 81: 272-283.

[2] Edwards MJ, Lemos FJ, Donnelly-Doman M, Jacobs-Lorena M (1997) Insect Biochem Mol Biol 27: 1063–1072.

[3] Clements AN (1992) *The biology of mosquitoes*, Chapman & Hall, London, New York.
